# Dental decision-making for persons with dementia: A systematic narrative review

**DOI:** 10.1097/MD.0000000000036555

**Published:** 2024-01-19

**Authors:** Shinpei Matsuda, Hitoshi Yoshimura

**Affiliations:** a Department of Dentistry and Oral Surgery, Unit of Sensory and Locomotor Medicine, Division of Medicine, Faculty of Medical Sciences, University of Fukui, Fukui, Japan.

**Keywords:** decision-making, dementia, dental, narrative review, systematic

## Abstract

Dental decision-making represents the establishment of a common understanding between the dental professional and the recipient of the intervention, which determines oral healthcare and dental treatment policies. Dental decision-making for persons with dementia can be challenging, and there have been no systematic reviews on this topic. Therefore, this systematic narrative review aimed to identify the current state of dental decision-making in persons with dementia. Literature search was performed using PubMed, Web of Science, Cochrane Library, CINAHL, and Google Scholar databases. Through the process of research selection, 7 articles with a high risk of bias were included in this study. This review clarified that there is limited information on the dental decision-making processes for persons with dementia. In conclusion, although this may be difficult due to different medical and socioeconomic conditions, the dilemma between the need to establish evidence for dental decision-making and medical ethics that prioritize a patient-centered position should be discussed globally in the future.

## 1. Introduction

Dementia is a chronic and progressive syndrome that results in cognitive deterioration beyond that expected during normal biological aging.^[1]^ Decision-making regarding the health, socio-economic environment, and personal information of persons with dementia requires the support of family members and various professionals.^[2–4]^ Family members and caregivers of persons with dementia face many important decision-making situations that require expertise and ethical considerations in various areas.^[5–7]^ Such situations usually occur suddenly, without any period of respite for consideration.^[5–7]^ Therefore, it is important for professionals to provide family member and caregivers of persons with dementia with extensive information to help them make prompt and appropriate decisions on the patients’ behalf.^[8]^

Dementia decreases a patient’s receptivity to oral health management and dental treatment and decreases their decision-making capability.^[9–12]^ Our previous studies have shown that older patients have multiple teeth with or without caries and periodontitis.^[13–15]^ These results suggest that when an older person has dementia, dental decisions are made by the family members or caregivers based on information provided by dental professionals.^[13–18]^ Although there is concern that dental decision-making for persons with dementia can be challenging in many situations, we believe that no valuable articles have systematically reviewed this issue. Moreover, we believe that the debate on dental decision-making for persons with dementia is becoming increasingly important as the world’s population ages.

This systematic qualitative review aimed to identify the current state of dental decision-making in persons with dementia.

## 2. Materials and methods

**Figure 1.**
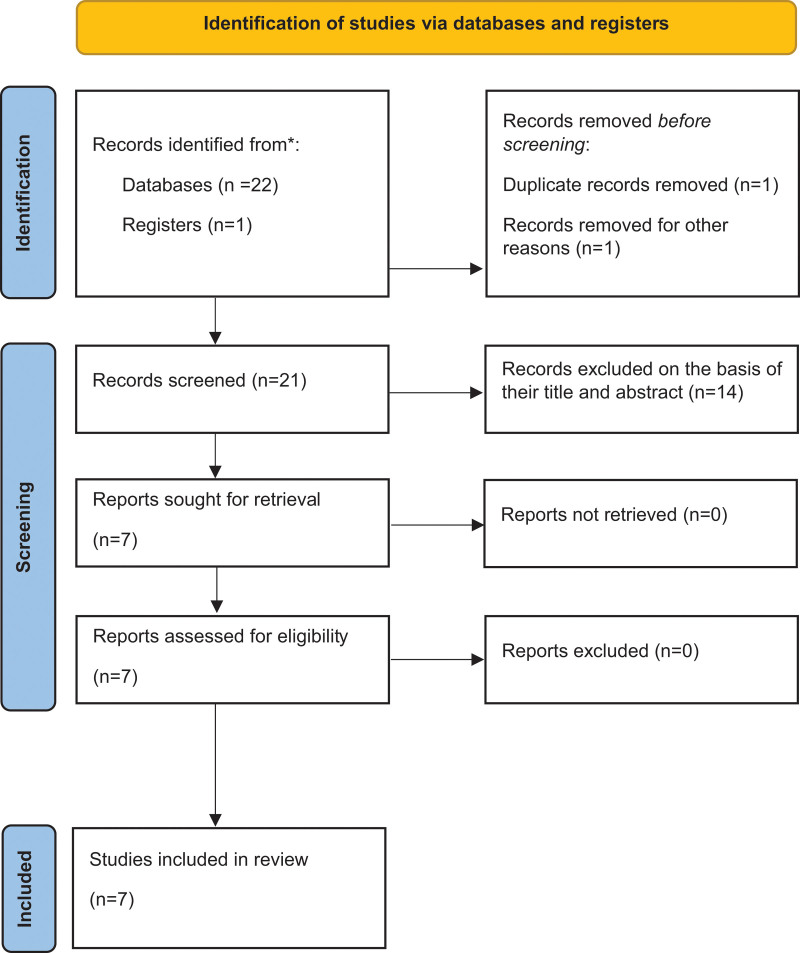
The flow diagram of Preferred Reporting Items for Systematic Reviews and Meta-Analyses 2020.

The systematic literature search was performed according to the guidelines, checklist, and flow diagram of the Preferred Reporting Items for Systematic Reviews and Meta-Analyses 2020 (Fig. 1).^[19–21]^

### 2.1. Inclusion and exclusion criteria

The inclusion criteria were as follows: (1) literature associated with dental decision-making for persons with dementia; (2) case reports, original articles, and review articles; and (3) literature published in English. Literature with unavailable full texts were excluded.

### 2.2. Information sources and literature search strategy

An electronic systematic literature search was conducted on October 16, 2022, using the PubMed, Web of Science, Cochrane Library, and CINAHL databases. The search included the following keywords: dental, decision-making, and dementia (Table 1). An additional manual search of Google Scholar using the same keywords was performed on the same day.

**Table 1:** The electronic literature search strategy.

Data base	Search strategy	Detected items
PubMed	((dental [MeSH Terms]) AND (decision-making [MeSH Terms])) AND (dementia [MeSH Terms])	4
Web of Science	(dental [Topic]) AND (decision-making [Topic]) AND (dementia [Topic])	9
Cochrane Library	(dental [Keyword]) AND (decision-making [Keyword]) AND (dementia [Keyword])	5
CINAHL	(dental [Subject]) AND (decision-making [Subject]) AND (dementia [Subject])	4

### 2.3. Study selection

The first author (S.M.) performed the electronic literature search and additional manual search, and all authors (S.M. and H.Y.) evaluated the studies based on the Preferred Reporting Items for Systematic Reviews and Meta-Analyses flow chart. Disagreements between reviewers were resolved through discussion and consensus.^[21]^

### 2.4. Risk of bias assessment

Considering concerns over quality and the risk of bias, the authors evaluated the studies based on the Critical Appraisal Skills Programme checklist for qualitative research, which consists of 10 questions.^[22]^

## 3. Results

### 3.1. Study selection

A total of 22 articles were identified (Fig. 1). After excluding a single duplicate article and 15 other articles based on their published language, titles, and abstracts, the remaining 6 articles were assessed for eligibility.^[9,23–27]^ Only 1 article was included through an additional manual search using the Google Scholar database.^[10]^ Finally, 7 studies were included in this systematic narrative review.

### 3.2. Synthesis of included studies

The 7 articles included in this systematic narrative review consisted of 2 case reports from the United States and Canada published in 2003 and 2014, respectively,^[23,24]^ 2 original articles from Sweden and Japan published in 1998 and 2005, respectively,^[9,25]^ and 3 review articles from the United Kingdom.^[10,26,27]^

### 3.3. Perspectives from case reports

Two articles on patients with dementia described the onset and progression of dementia and identified the following dental problems: poor oral hygiene, dental caries, periodontitis, peri-implantitis, and problems associated with dental prostheses including dentures and crowns.^[23,24]^ These cases required dental decision-making by third parties such as family members, with the support and suggestions of the dentist involved.^[23,24]^ These articles reported the potential for compromised dental treatment and oral hygiene management.^[23,24]^

### 3.4. Perspectives from original articles

A Swedish study reported that dentists treating patients with dementia faced a moral dilemma from conflicting perspectives associated with restraints on public funds and the ethical duty to respect humanity.^[9]^ Another study from Japan reported that the cognitive status of patients with dementia was negatively associated with denture acceptance and use; thus, these results should be considered in the decision-making process regarding denture treatment.^[25]^

### 3.5. Perspectives from review articles

An article from the United Kingdom published in 2007 reported that dental professionals were required to comply with the Mental Capacity Act 2005 enacted in the United Kingdom when treating patients without dental decision-making capacity.^[26]^ According to a 2018 article from the United Kingdom, although guidelines and acts were available to assist in decision-making for patients who may lack mental capacity, informed consent such as the risks and benefits of both treating and deciding not to treat remained crucial in determining the most appropriate approach to decide the dental treatment policy.^[27]^ In 2020, another article from the United Kingdom reported that dental decision-making for patients with dementia depended on dental conditions, risks and benefits of the treatment under consideration, stage of dementia, and ability to comply.^[10]^ It is necessary to consider the patients’ health and well-being, including social and cultural factors in planning their care.^[10]^

### 3.6. Risk of bias assessment

The authors assessed the risk of bias of the 7 included articles using the Critical Appraisal Skills Program checklist for qualitative research.^[22]^ Based on these assessments, we concluded that this systematic narrative review had a high risk of bias because it included both case reports and reviews.

## 4. Discussions

This systematic narrative review clarified that there is limited information on dental decision-making for persons with dementia, leaving room for discussion. In addition, we believe that this review is clinically valuable, as it reveals the evolution of this debate over the past 20 years. Interestingly, this study clarified that there has been a long debate and repeated reconsideration from the patient’s perspective regarding dental decision making, especially in the United Kingdom.^[10,26,27]^ Although this may be difficult because of different medical and socioeconomic conditions, the authors believe that the direction of dental decision-making for persons with dementia should be discussed globally in the future. These efforts have the potential to reduce the emotional stress experienced by family members, caregivers, and others involved in the dental decision-making process for persons with dementia.

Previous studies on dental decision-making have reported that a variety of clinical and nonclinical factors are involved.^[16,28–36]^ These articles included the following clinical factors: best available evidence and tooth-related factors, such as tooth condition with caries and lesions, and past treatment status.^[16,28–36]^ Additionally, these studies considered non-clinical factors such as dentists’ background (age, specialty area, education, and mental status) and dental practice management status.^[16,28–36]^ The authors believe that previous studies have focused on the factors of dental professionals and the effects of objective and clinical dental information on dental decision-making, while there has been insufficient research on the effects of patients’ mental status, such as dementia, on dental decision-making.

A study from Brazil in 2005 showed that patients’ race may influence dental decision-making.^[37]^ In 2022, Vianna et al reported that socioeconomic status was significantly involved in dental decision-making in Brazil.^[38]^ Currently, this situation may have become more critical, owing to the COVID-19 worldwide pandemic since 2019, which has exacerbated the existing racial/ethnic and socioeconomic disparities in public health.^[14,39,40]^ Socioeconomic status and dementia are closely related and may be important factors in dental decision-making for persons with dementia.^[41–45]^ Dentists must achieve the Sustainable Development Goals proposed by the United Nations through ethical and evidence-based dental practice.^[46–49]^

The present study has the following limitations: (1) it included relatively few articles; (2) it included case reports and review articles; and (3) the study regions/research areas of the included articles contributed to bias in the present study. The authors conclude that this review has significant limitations and biases. Therefore, the authors believe that further high-quality research is needed on dental decision-making for persons with dementia.

The debate over the past 2 decades in the United Kingdom may be indicative of the evolution of perspectives on the personal dignity and medical ethics of persons with dementia.^[10,26,27]^ We believe that ethical considerations have influenced and hindered the promotion of clinical research in this area. The authors conclude that the dilemma between the need to establish evidence based on clinical trials for dental decision-making in persons with dementia and medical ethics that prioritizes a patient-oriented position should be discussed.

## 5. Conclusion

This systematic narrative review clarified that there is limited information on the dental decision-making process for persons with dementia. Therefore, the dilemma between the need to establish clinical evidence for dental decision-making and medical ethics prioritizing a patient-centered position should be discussed globally in the future.

## Author contributions

**Conceptualization:** Shinpei Matsuda.

**Data curation:** Shinpei Matsuda.

**Writing – original draft:** Shinpei Matsuda.

**Writing – review & editing:** Shinpei Matsuda, Hitoshi Yoshimura.
